# Using Biotic Interaction Networks for Prediction in Biodiversity and Emerging Diseases

**DOI:** 10.1371/journal.pone.0005725

**Published:** 2009-05-28

**Authors:** Christopher R. Stephens, Joaquín Giménez Heau, Camila González, Carlos N. Ibarra-Cerdeña, Victor Sánchez-Cordero, Constantino González-Salazar

**Affiliations:** 1 C3 - Centro de Ciencias de la Complejidad, Universidad Nacional Autónoma de México, Ciudad de México, México; 2 Instituto de Ciencias Nucleares, Universidad Nacional Autónoma de México, Ciudad de México, México; 3 Instituto de Biología, Universidad Nacional Autónoma de México, Ciudad de México, México; BMSI-A*STAR, Singapore

## Abstract

Networks offer a powerful tool for understanding and visualizing inter-species ecological and evolutionary interactions. Previously considered examples, such as trophic networks, are just representations of experimentally observed direct interactions. However, species interactions are so rich and complex it is not feasible to directly observe more than a small fraction. In this paper, using data mining techniques, we show how potential interactions can be inferred from geographic data, rather than by direct observation. An important application area for this methodology is that of emerging diseases, where, often, little is known about inter-species interactions, such as between vectors and reservoirs. Here, we show how using geographic data, biotic interaction networks that model statistical dependencies between species distributions can be used to infer and understand inter-species interactions. Furthermore, we show how such networks can be used to build prediction models. For example, for predicting the most important reservoirs of a disease, or the degree of disease risk associated with a geographical area. We illustrate the general methodology by considering an important emerging disease - Leishmaniasis. This data mining methodology allows for the use of geographic data to construct inferential biotic interaction networks which can then be used to build prediction models with a wide range of applications in ecology, biodiversity and emerging diseases.

## Introduction

A fundamental underlying goal of biology is to model the distribution of biota and identify their interactions, thus permitting both an understanding of current distributions and the possibility of predicting future ones [1]. Such models have important applications, such as in biodiversity [2] and emerging diseases. Networks offer an important tool for understanding and visualizing biotic interactions and have been used in a variety of contexts [4], [5], [6]. They are constructed by linking nodes of the network, usually species that have a known interaction, such as in trophic webs. However, as it is not feasible to exhaustively track the large numbers of ecological interactions, the question arises: can biotic interaction networks be constructed other than by direct observation, using other available data?

There is evidence that the evolutionary dynamics of inter-species interactions create rich geographic mosaics [7]. Moreover, phylogenetic research has shown that species are conservative when it comes to the taxa with which they interact, both spatially and temporally. As an example relevant to this paper, blood sucking insects have evolved phenotypic traits to optimize host-seeking and feeding [8]. Co-distributions of host and parasite will then reflect the strong biotic relation that exists between them. Similarly, as a reflection of the potential confrontation of species, co-occurrence could also engender an interaction in the absence of a pre-existing one [9]. We are thus led to consider distributional data for constructing inter-species interaction networks.

Collection data offer an important proxy for modeling distributions. Here, we show how such data can be used to infer potential inter-species interactions, construct an associated network and, further, show how that network can be used to construct prediction models. Collection data are already widely used in biodiversity informatics [10], [11], and have been principally used for constructing species distributions from abiotic niche variables only. The data are taxonomic in nature and georeferenced, the set of point collections of a species in a geographical region giving a sampling for the distribution of the species in that region. Of course, there is an important question of sample bias in the data [12], [14] (see also the Materials and Methods section), though its extensive use and utility, even in areas where data are scarce [13], is testament to the fact that it can yield important information if treated carefully. Additionally, in the case of urgent problems of great social impact, such as that of emerging diseases, it is important to try to leverage the data that actually exist, at least until better, more bespoke, data become available.

## Results

Dividing up a geographic region into spatial cells, x_α_, we take as our underlying variable of interest, B_i_(x_α_), a measure of the distribution of the ith taxon in the spatial cell x_α_. The specific form of B_i_ is determined by the available data - relative or absolute abundance, presence/absence or presence only. A fundamental object of interest is P(B_i_(x_α_)|**I**(x_α_)), the probability that the distribution measure B_i_(x_α_) takes a certain value in the spatial cell x_α_ conditioned on, **I**(x_α_), which is composed of, in principle, all biotic and abiotic variables that affect species distributions, and which constitute the biotic and abiotic profiles of the corresponding niche [10]. An example of interest would be that B_i_(x_α_) represents presence of the ith species in the spatial cell x_α_.

As we have no underlying theory with which to construct P(B_i_(x_α_)|**I**(x_α_)) we will use a data mining approach to estimate it, using point collection data as a proxy for the actual distribution of taxa. Point collection data here represent the set of georeferenced localities (latitude, longitude and date) of museum voucher specimens. It is important to remember that the distribution of taxa is a direct result of the past and present interactions of all relevant causative factors - climactic, phylogenetic, co-evolutionary, ecological etc. Hence, part of the task of any analysis is to determine, out of the myriad of factors that contribute to **I**, which ones are the most predictive in determining a particular distribution. An immediate problem is that, as every spatial cell is unique, for each x_α_ one has a statistical sample of size one and hence P(B_i_(x_α_)|**I**(x_α_)) = 0 or 1.

To overcome this, one first constructs the relationship between B_i_ and **I**, such as P(B_i_| **I**), via a sampling of all spatial cells in order to obtain the relationship between a given distribution measure and the associated niche variables. With this in hand, a “profile” of any given spatial cell x_α_ can be constructed in terms of the biotic and abiotic niche variables and the relationship between B_i_ and **I** used to determine P(B_i_(x_α_)|**I**(x_α_)) (see Materials and Methods section).

As P(B_i_| **I**) involves counting the number of spatial cells where there is a co-occurrence of the ith species with a particular configuration of the niche variables **I**, if **I** is of high dimension then the number of cells where there are co-occurrences will be small or zero. We thus restrict attention for the moment to the case where **I** is a single variable, I_j_, so that 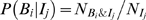, where 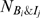 is the number of cells with a co-occurrence of the distribution variable B_i_ and the niche variable I_j_, and 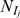 is the number of cells with niche variable Ij. In the case where Ij is also a taxon distribution, and we consider presence, then P (B_i_ |B_j_) measures the probability of presence of taxon B_i_ given the presence of taxon B_j_ and is thus a measure of the statistical association between B_i_ and B_j_. As P (B_i_ |B_j_) does not take into account statistical confidence however, we consider rather
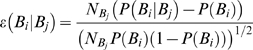
which also measures the degree of confidence one can have in the statistical association between B_i_ and B_j_ relative to the null hypothesis, P(B_i_), that the distribution of B_i_ is independent of B_j_ and distributed with this probability over the region of interest. Essentially, this is a one-sided binomial test where the null hypothesis is that the distribution of B_i_ is random over the sample space; in this case the cells of the region of interest. It can, of course, be useful to consider other null hypotheses. For instance, one could use as null hypothesis P(B_i_| **A**) where **A** represents a set of abiotic factors, or the result of a niche-model such as GARP or MaxEnt [15], [16]. Values of |ε(B_i_ |B_j_)| greater than a certain threshold (see Materials and Methods section) measure the degree to which the data is consistent with the null hypothesis. In the case where the binomial distribution associated with P(B_i_) can be approximated by a normal distribution then values of |ε(B_i_ |B_j_)|>2 would indicate an inconsistency between the data and the null hypothesis to the 95% confidence level.

For any pair of taxa, B_i_ and B_j_, taken as nodes of a network, a link between them, whose “strength” is given by ε(B_i_ |B_j_), or P (B_i_ |B_j_), can be graphed. The resulting interaction network then offers a visualization of the inferred statistical dependencies between different taxa. Note that, contrary to networks that are common in the literature, that represent known interactions, such as between predator and prey in a trophic web [15], this network represents statistical associations from which inferences about real causal interactions can be made and then tested. Higher order statistical associations, such as P (B_i_ |B_j_ B_k_), can also be examined. Such an interaction could be represented by three nodes, with links from B_j_ and B_k_ to B_i_, and would represent the degree of statistical dependence of taxon B_i_ on the co-occurrence of the taxa B_j_ and B_k_. From the network, for a given node B_i_, a ranked list of values of ε(B_i_ |B_j_), or P (B_i_ |B_j_), can be taken as a model for predicting the most important potential biotic interactions of the species B_i_. To determine P (B_i_ |**I** ^′^) when **I** ^′^ is high dimensional, a statistical model must be used to approximate it. A very useful and transparent one, that can be deduced using only the properties of the network, is the naive Bayes approximation [22] (see the Materials and Methods section), wherein a score function, S (B_i_ | **I′**), that is a monotonic function of P (B_i_ | **I** ^′^), can be constructed. The score consists of a sum of contributions from each niche variable, both biotic and abiotic, from which it is possible to observe which are the most important niche variables.

As an example of the general methodology we consider an important emerging disease - Leishmaniasis - a vector borne disease widely distributed in tropical regions that is estimated to affect 12 million people in 88 countries. Since Leishmaniasis is a zoonotic tropical disease, sylvan reservoirs are crucial to the maintenance of the parasite in ecological communities and, further, are intimately associated with human transmission [18]. Reservoirs of *Leishmania* can be classified as primary and incidental, according to their importance in the long-term transmission of the parasite, being considered incidental if they are dead ends that do not transmit to vectors [19]. Although direct experiment could determine to which type a given reservoir belongs, when there are many potential reservoirs other alternatives, such as that presented here, are more feasible.

**Figure 1:**
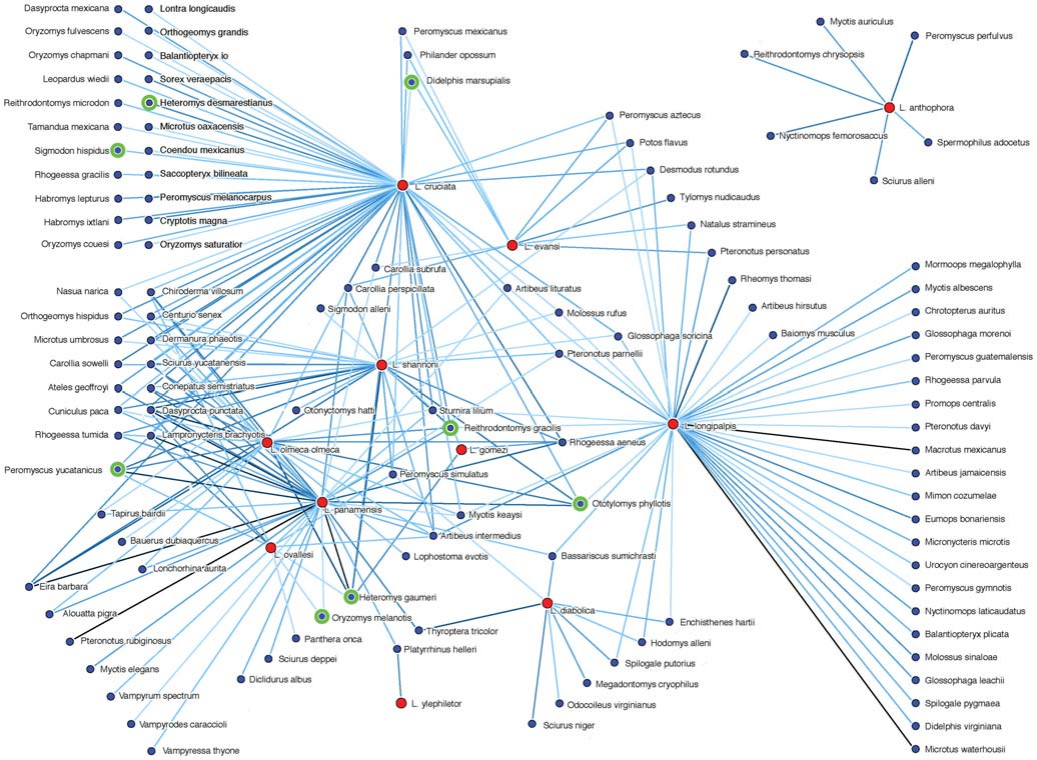
Interaction network between potential and confirmed vectors and reservoirs for *Leishmania* in Mexico. Mammal species confirmed as reservoirs for *Leishmania mexicana*, responsible for the cutaneous form of the disease are marked with a double circle. One species, *Didelphis marsupialis* is the known sylvatic reservoir for the visceral form.

We used collection data points for 427 terrestrial mammal species occurring in Mexico as potential or confirmed reservoirs and 11 species of *Lutzomyia* as confirmed or potential vectors for *Leishmania*. The description of the data set can be found in the Materials and Methods section. *Lutzomyia* is a genus of “sand flies” that in the New World is responsible for the transmission of the *Leishmania* parasite. Only females suck blood for egg production. In Mexico there is little information about which vectors are involved in transmission of the parasite in different geographic regions. The only confirmed vector is *Lutzomyia olmeca olmeca* [20]. However, several species have been found with the parasite - *Lutzomyia olmeca olmeca*, *Lutzomyia cruciata* and *Lutzomyia ovallesi* [21]. With respect to transmission of the visceral form of the disease the principle vector *Lutzomyia longipalpis* has been collected in Mexico but has not been reported with the parasite. For the secondary vector *Lutzomyia evansi*, there exists only one collection in the state of Chiapas which was without infection [22]. In Mexico, there are only eight mammal species found infected with *Leishmania mexicana* parasites, responsible for the cutaneous form of the disease, identified in the state of Campeche in southern Mexico [23], [24], [25]; a very small number when compared to the total number of potential reservoirs. It is important, therefore, to be able to predict which currently unidentified mammals are most likely to be important as actual or potential reservoirs for the disease. As a measure of statistical association we consider P(v_i_ |m_j_) and ε(v_i_ |m_j_), where v_i_ represents the ith vector and m_j_ the jth potential reservoir. There are 4697 potential vector-reservoir pairs. In Figure 1 we show the 241 most important positive associations (highest values of ε) between *Lutzomyia* as vectors and mammals as suspected and confirmed reservoirs for *Leishmania*. The vector species are marked as red nodes, while the confirmed reservoirs are marked as green. The darker the link, the stronger is the associated statistical dependence between the associated *Lutzomyia* and mammal.

The connectivity of the network is related to the geographical distribution of the different species and has consequences for the way in which a parasite could propagate across the network from one geographical region to another. The separated subnetwork corresponds to *L. anthophora*, a species indigenous only to the north of Mexico and the United States. For *Lutzomyia* nodes, the vertex degree dictates with how many mammals a given vector shares important positive statistical associations, while, for mammal nodes, the vertex degree tells us how many vectors are potentially exploiting the mammal. A high vertex degree for a given vector shows that it could potentially exploit many different mammals. Moreover, if there are many connections to mammals that are not connected to other vectors, then all else being equal, it would be evolutionarily suboptimal for the vector not to exploit them. *L. cruciata* and *L. longipalpis*, in particular are associated with large numbers of mammals that have no statistical relation with other vectors. On the other hand, *L. olmeca*, *L. ovalesi*, *Lutzomyia shannoni* and *Lutzomyia panamanensis* are all within a highly connected part of the network that corresponds geographically to the peninsula of Yucatan, where many mammals are associated with several different vectors. In such circumstances, a vector may adopt a strategy of specializing to a smaller group of species in order to avoid competition. Interestingly, four of the eight infected rodent reservoirs - *Peromyscus yucatanicus*, *Ototylomys phyllotis*, *Reithrodontomys gracilis* and *Heteromys gaumeri*, all restricted to the peninsula of Yucatan, have very high vertex degrees, a fact that associates them with higher risk, as potentially many different vector species can exchange parasites with them.

Besides offering substantial insight into the ecological interactions between potential vectors and reservoirs of a disease, the interaction network can also be used to obtain predictive models. Here we consider two such models - one for directly predicting the most important potential disease reservoirs and another for predicting a measure of disease risk for a given geographic area. Turning first to the prediction of potential reservoirs: with ε(v_i_ |m_j_) in hand, for a given vector v_i_, we can construct a ranked list, from maximum to minimum value, of ε(v_i_ |m_j_), over all pairs (v_i_,m_j_), i.e., a ranking of the links of a given node according to their strength. Those mammals with the highest values of ε are predicted to correspond to the most important potential reservoirs for that vector. In Table 1 we show the results for the highest 150 values of ε(v|m_j_), where to obtain the list we have grouped together the different *Lutzomyia* species into one group v to form a list of 427 values of ε(v|m_j_) as a function of j. The highest ranked mammals have the highest degree of statistical correlation with *Lutzomyia*, with the implication that these mammals are the most important potential reservoirs for *Leishmania*. By grouping together the different *Lutzomyia* species we are considering association between a given mammal species and the different species of the *Lutzomyia* genus present in Mexico, rather than with individual species, thus increasing the sample size and allowing for more robust statistics. A secondary logic for this is also that the biomass of parasite that can pass from vector to mammal in a given spatial cell depends on the number of different vector species that are present in that cell. Thus, a mammal with a high probability of co-occurrence with more than one *Lutzomyia* will, all else being equal, present a higher degree of risk of having the *Leishmania* parasite transmitted to them than one that has a high degree of occurrence with only one species.

**Table 1:** Ranked list of potential mammal reservoirs for *Leishmania* in Mexico.

	Mammals	Epsilon	Conf.		Mammals	Epsilon	Conf.		Mammals	Epsilon	Conf.
1	Eira barbara	10.1683		51	Molossus sinaloae	5.8518		101	Balantiopteryx plicata	3.8590	
2	Rhogeessa aeneus	9.3649		52	Artibeus lituratus	5.8422		102	Peromyscus leucopus	3.7994	
3	Artibeus intermedius	9.1628		53	Mormoops megalophylla	5.8374		103	Sturnina ludovici	3.7888	
4	Reithrodontomys gracilis	8.8921	Yes	54	Habromys lepturus	5.7848		104	Enchisthenes hartii	3.6929	
5	Carollia sowelli	8.8303		55	Myotis keaysi	5.6148		105	Vampyrodes caraccioli	3.6929	
6	Heteromys gaumeri	8.8000	Yes	56	Chiroderma villosum	5.5562		106	Eptesicus furinalis	3.6453	
7	Peromyscus mexicanus	8.7859		57	Tamandua mexicana	5.4845		107	Liomys pictus	3.6107	
8	Heteromys desmarestianus	8.7164	Yes	58	Tylomys nudicaudus	5.4510		108	Glossophaga commissarisi	3.4861	
9	Molossus rufus	8.6277		59	Saccopteryx bilineata	5.2984		109	Lonchorhina aurita	3.4781	
10	Glossophaga soricina	8.5713		60	Macrotus mexicanus	5.2472		110	Phyllostomus discolor	3.4781	
11	Carollia perspicillata	8.5030		61	Sciurus aureogaster	5.2267		111	Peromyscus gymnotis	3.4516	
12	Orthogeomys hispidus	8.3468		62	Baiomys musculus	5.2092		112	Anoura geoffroyi	3.4201	
13	Pteronotus parnellii	8.1632		63	Rhogeessa tumida	5.1950		113	Platyrrhinus helleri	3.3586	
14	Desmodus rotundus	8.1519		64	Sciurus deppei	5.1414		114	Eumops bonariensis	3.3398	
15	Dasyprocta mexicana	8.1128		65	Dermanura watsoni	5.1338		115	Sciurus variegatoides	3.3398	
16	Sturnira lilium	8.0290		66	Otonyctomys hatti	5.1338		116	Uroderma bilobatum	3.3373	
17	Dermanura phaeotis	8.0055		67	Orthogeomys grandis	5.0556		117	Lasiurus intermedius	3.2197	
18	Dasyprocta punctata	7.9678		68	Alouatta palliata	5.0457		118	Lasiurus ega	3.1739	
19	Oryzomys couesi	7.7253		69	Choeroniscus godmani	5.0457		119	Peromyscus megalops	3.1410	
20	Potos flavus	7.7246		70	Peropteryx macrotis	5.0457		120	Eumops glaucinus	3.0564	
21	Conepatus semistriatus	7.6879		71	Pteronotus personatus	5.0266		121	Urocyon cinereoargenteus	2.9697	
22	Ototylomys phyllotis	7.5587	Yes	72	Lontra longicaudis	4.9330		122	Procyon lotor	2.9502	
23	Ateles geoffroyi	7.4787		73	Reithrodontomys mexicanus	4.9120		123	Hylonycteris underwoodi	2.9343	
24	Cryptotis magna	7.4207		74	Oryzomys rostratus	4.8681		124	Rhynchonycteris naso	2.8580	
25	Cuniculus paca	7.3220		75	Mimon cozumelae	4.8327		125	Eptesicus brasiliensis	2.8106	
26	Lampronycteris brachyotis	7.2852		76	Pteronotus davyi	4.7943		126	Myotis albescens	2.8106	
27	Sigmodon hispidus	7.2805	Yes	77	Herpailurus yagouaroundi	4.7100		127	Lophostoma evotis	2.8106	
28	Peromyscus yucatanicus	7.2486	Yes	78	Glossophaga leachii	4.6849		128	Tapirus bairdii	2.8106	
29	Oryzomys chapmani	7.1242		79	Rhogeessa gracilis	4.6317		129	Vampyrum spectrum	2.8106	
30	Didelphis virginiana	7.1150		80	Sylvilagus brasiliensis	4.6317		130	Marmosa mexicana	2.7731	Yes
31	Peromyscus melanocarpus	7.0260		81	Hodomys alleni	4.5155		131	Peromyscus furvus	2.7731	
32	Microtus umbrosus	6.9630		82	Leopardus wiedii	4.4420		132	Myotis velifera	2.5757	
33	Thyroptera tricolor	6.9630		83	Peromyscus simulatus	4.4195		133	Spilogale putorius	2.5411	
34	Nasua narica	6.8953		84	Sigmodon alleni	4.3707		134	Microtus mexicanus	2.5268	
35	Megadontomys cryophilus	6.6830		85	Bassariscus sumichrasti	4.3110		135	Dasypus novemcinctus	2.4725	
36	Oryzomys alfaroi	6.6816		86	Oryzomys fulvescens	4.3110		136	Myotis nigricans	2.4704	
37	Sorex veraepacis	6.6797		87	Diphylla ecaudata	4.3013		137	Lophostoma brasiliense	2.4407	
38	Carollia subrufa	6.6316		88	Oryzomys melanotis	4.2907	Yes	138	Diclidurus albus	2.4407	
39	Peromyscus aztecus	6.6173		89	Micronycteris microtis	4.2338		139	Sciurus niger	2.4407	
40	Didelphis marsupialis	6.4390	Yes	90	Mazama americana	4.2274		140	Leptonycteris curasoae	2.4268	
41	Sciurus yucatanensis	6.3865		91	Microtus oaxacensis	4.2061		141	Nyctomys sumichrasti	2.4026	
42	Philander opossum	6.2546		92	Rheomys thomasi	4.2061		142	Sigmodon mascotensis	2.3815	
43	Habromys ixtlani	6.1120		93	Oryzomys saturatior	4.2061		143	Alouatta pigra	2.3374	
44	Microtus waterhousii	6.1120		94	Myotis elegans	4.2024		144	Peromyscus melanophrys	2.2204	
45	Pteronotus rubiginosus	6.1120		95	Oligoryzomys fulvescens	4.1984		145	Dermanura tolteca	2.1920	
46	Reithrodontomys microdon	6.0967		96	Natalus stramineus	4.0626		146	Trachops cirrhosus	2.1663	
47	Coendou mexicanus	6.0268		97	Balantiopteryx io	4.0522		147	Bauerus dubiaquercus	2.1612	
48	Centurio senex	6.0076		98	Nyctinomops laticaudatus	4.0522		148	Spilogale pygmaea	2.1612	
49	Artibeus jamaicensis	5.9786		99	Tlacuatzin canescens	4.0119		149	Leptonycteris nivalis	2.1402	
50	Glossophaga morenoi	5.8847		100	Odocoileus virginianus	3.9265		150	Sylvilagus floridanus	2.1002	

Such a ranked list provides a general model for predicting the most important likely reservoirs for any given disease. Note that, of the eight infected reservoirs of *Leishmania* in Mexico, six of them, including the four confirmed, appear in the top 7% of ranked predictions of most important potential reservoirs. If we take as null hypothesis that the confirmed reservoirs are distributed randomly in the ranked list, then the probability that they appear with their actual rankings is less than 10^−8^, thus showing that the model's results are statistically significant and that the model predicts very well, especially given the relative lack of information on which it is based, in that at no point was information on confirmed reservoirs used to “train” the model. Of course, one could argue that, all else being equal, there should be a higher degree of co-occurrence between *Lutzomyia* species and those mammals that are most widespread, as these will have had a higher probability of having being tested as potential reservoirs. Of course, if this were true, it would greatly reduce the predictive power of the model. We tested this hypothesis on a subset of 360 mammal species where distribution data was readily available. The positions of the confirmed reservoir species ranked according to their area of distribution were: 25, 152, 154, 200, 224, 230, 249, 255 and 257; while when ranked according to our prediction model the positions were 4, 6, 8, 22, 27, 28, 40, 88 and 130. As a simple statistical comparison one can compare the mean rank from both methods using an independent two sample t-test. The test statistic value is 5.4 corresponding to a p value of less than 0.001 clearly indicating that the predictive power of our model cannot be explained by assuming that those species with larger distributions are more likely to be confirmed reservoirs.

The third step we will take is to construct a predictive model to quantify disease “risk” in any given geographic cell. Here we take as risk measure the probability that disease vectors are present, while the prediction itself is based only on biotic factors, i.e., the presence of potential mammal reservoirs. Explicitly, a score function, S (B_i_ | **B**), for predicting class membership is constructed, where B_i_ is associated with the ith vector species and **B** represents the presence of mammal species, B_1_, …, B_N_, and related to the posterior classifier probabilities, P (B_i_| **B**), using the naive Bayes approximation, 
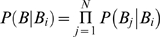
, the factors P(B_j_ |B_i_) being associated with directed links from vector to reservoir in the network. The advantage of this approximation is that the contribution of each biotic niche variable, B_i_, is independent of the rest, so that, in the case where abiotic variables are also explicitly included, the relative importance of both biotic and abiotic factors can be studied. As one would expect in the present case, biotic variables play a more important role than abiotic ones, due to the direct dependence of a vector on its associated reservoirs. With S(**B**) in hand, the biotic niche profile of any geographical area can be determined using a ranked list of niche characteristics and allows one to see at a glance which species are playing an important role.

**Figure 2:**
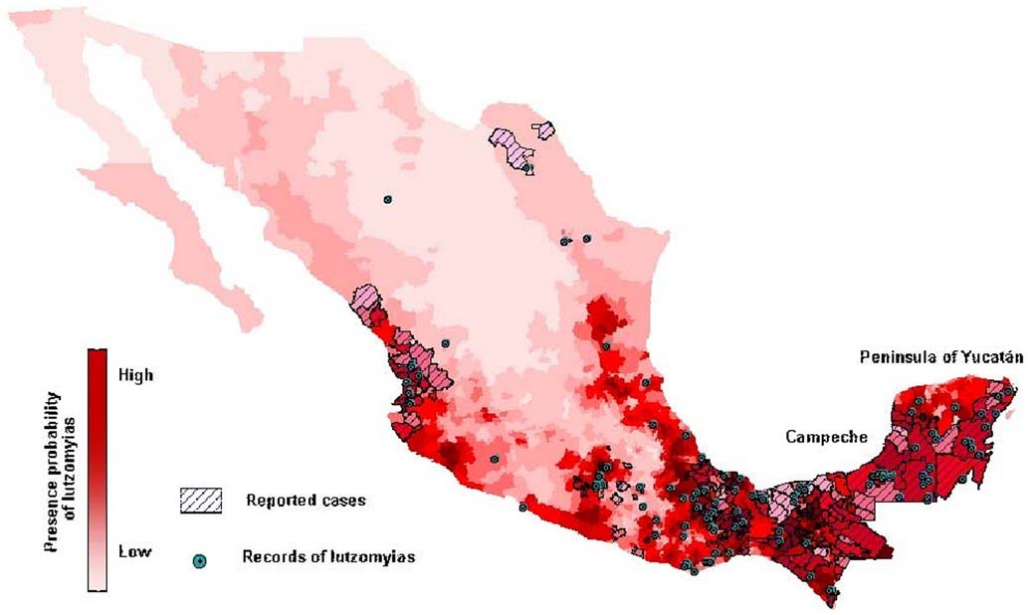
Biotic risk map for *Leishmania* using the mapped score function.

In Figure 2 we see the results for the grid partition of Mexico we used earlier. The redder/whiter the area the higher/lower the predicted probability for finding *Lutzomyia* based only on co-occurrence with mammal species, the mid-range being associated with the probability P(B_i_) associated with the null hypothesis. Also shown is the georeferenced set of point collections of *Lutzomyia*. As can be seen, the agreement is good, though there are one or two outliers. Finally, on the map we also see those geographical regions where cases of *Leishmania*sis have been reported. The shaded regions correspond to “municipios” (municipalities) where *Leishmania*sis cases have been reported in the last 40 years. Note that the area of different municipios can vary greatly. In regions where there is no cross-hatching there are no cases that have been reported to the Secretaria de Salúd Pública (Governmental Public Health Agency) in Mexico. This does not necessarily imply that there are none, as there is no obligatory reporting of cases of Leishmaniasis in Mexico. In this sense reported cases are the equivalent of presence data, while no reported cases does not imply “absence”. A noteworthy feature of the map is that there are no areas with reported cases where the model does not predict a higher than random probability for presence of *Lutzomyia*. In interpreting the apparent overprediction several comments are in order: First of all, as mentioned, the quality of reporting data of cases of *Leishmania*sis varies significantly from state to state in Mexico. Secondly, the map is of degree of risk due to biotic factors only; the output being a score that measures the probability of *Lutzomyia* being present in a given spatial cell. In that sense, it is a map associated with only one type risk factor, all be it an important and necessary one for the presence of the disease in the human population which, obviously, depends on many other factors. By including such factors, for example, abiotic or socio-demographic variables, more complex risk models can be simply created using our methodology.

## Discussion

The main contribution of this paper is to show how biotic interaction networks may be constructed inferentially using a data mining approach applied, in this case, to point collection data, rather than by direct observation, and to show that these networks can be used, not only to understand and visualize potential inter-species interactions, but also to formulate prediction models. The important area of emerging diseases was used as a test bed to show the utility of the approach. The main logic of this methodology is that current distributions of biota, as proxied by point collection data for the example given here, adequately reflect all causal influences, both biotic and abiotic. The task, for a given set of input variables, is to discriminate which ones are of greater influence for a particular distribution. In this paper we used only biotic variables. A statistical dependence between two species infers, but does not prove, a direct biotic causal relationship. Thus, for a pair of nodes the strength of the link between them measures the degree to which two species tend to co-occur. If they co-occur in a statistically significant way we are prompted to identify as a plausible explanation a vector-reservoir interaction.

In the case of *Lutzomyia* and mammals this understanding comes from the natural potential direct causal relationship there: that the *Lutzomyia* feed on the corresponding mammal. The properties of the corresponding biotic network show to what extent a given vector is exploiting its potential food sources, evolutionary dynamics giving a logic as to why this usage should be optimal. From the network, the corresponding list of predicted reservoirs for a given *Lutzomyia* is not based on the physiological possibility that a given mammal is a reservoir but, rather, on the fact that a mammal with a high fraction of co-occurrences is more likely to be an important food resource for *Lutzomyia* than one with a small fraction and, therefore, that there is greater transmission of the parasite from one to the other. Moreover, as ε(v_i_ |m_j_) increases as the range of the mammal m_j_ grows, then this measure also predicts the degree of importance of the reservoir, a reservoir of small range being of less potential impact, all else being equal, than one of ample range. As mentioned, the utility of the model is clearly in evidence, given that all known reservoirs in Mexico are highly ranked in the complete list of 427 possible candidates.

To create spatial prediction models we used a model that utilized only information that came from the biotic interaction network. The associated score is a measure of the probability that *Lutzomyia* are present, which we can take as a proxy for the probability that the disease is present. To relate this to the number of cases in a more sophisticated model would require the inclusion of socio-economic and socio-demographic variables among others.

The results of this paper clearly lead us in the direction of making corresponding hypotheses that can be verified by further empirical research. Our ranked list of potential reservoirs is, as emphasized, based on the relative importance of the potential reservoir in terms of what biomass of parasite can potentially be harbored in a given spatial cell rather than what mammals are physiologically capable of being reservoirs. To test this, the following scenario may be envisaged: consider the known distribution of a given mammal from the list; select spatial cells at random from this distribution; in each cell capture the chosen mammal species and test for the presence of *Leishmania*. The appropriate metric is the proportion of spatial cells in which specimens were found with the parasite or, alternatively, if sufficient statistics may be obtained, ε(cells with specimens with parasite | total cells with specimens). This would be repeated for different mammal species. The hypothesis is that a highly ranked species will yield higher values for these two metrics than a low ranked one. To facilitate testing the hypothesis, the most appropriate species would be those chosen from different points in the ranked list that are common in a given geographical region and easy to capture. Of course, many mammals simply do not have any geographical overlap with the vectors. Strictly speaking one should consider these mammals too and test for presence of the parasite. Common sense would dictate that for those species far away from the known distribution of the vectors there is effectively zero probability of finding the parasite thus obviating the need to explicitly check these areas. Work is currently being planned to undertake these tests.

## Materials and Methods

The data set consisted of point collection data associated with one Class, Mammalia, and one genus - *Lutzomyia*. The mammal data set consisted of 37,297 point collections from georeferenced localities for 427 terrestrial mammals occurring in Mexico. The data were obtained from museum voucher specimens from national and international museum collections, public electronic databases (MaNIS; www.manis.gob.mx, and CONABIO; www.conabio.gob.mx) and published records [27], [28]. For *Lutzomyia*, there were 270 point collections, taken from published literature and from national entomological collections (Instituto de Diagnóstico y Referencia Epidemiológica (InDRE, Mexico City), the Colección Entomológica Regional Universidad Autónoma de Yucatán (UADY, Mérida) and the Laboratorio de Medicina Tropical at the Universidad Nacional Autónoma de México (UNAM, Mexico City), associated with 11 species. For both data sets, each locality was georeferenced to the nearest 0.01 degrees of latitude and longitude using 1∶250,000 topographic maps (INEGI; www.inegi.gob.mx, Instituto de Geografía, Universidad Nacional Autónoma de México; www.igeograf.unam.mx). Point collection data was, of course, not collected in order to provide an unbiased sampling of underlying species abundance and therefore must be considered carefully to understand potential statistical biases that might be present. The utility and limitations of point collection data have been amply discussed in [12], [14].

With respect to the data set for Mexican mammals, this data has been collected over a period of more than 100 years with a consequently large number of collectors [24], [25]. Hence, although the data has not been collected systematically, it has probably led to an adequate sampling. Additionally, mammals are the best known and collected group in Mexico. In the case of *Lutzomyia* the coverage is less but still represents the best available. With the registered cases of Leishmaniasis, unfortunately, there is no compulsory reporting of these in Mexico. So one can infer where the disease is present but not where it is absent. In problems of great social impact, such as that of emerging diseases, it is important to try to leverage the data that actually exists, at least until better more bespoke data becomes available. Parasite detection studies in potential reservoirs have been carried out principally in the state of Campeche. Van Wynsberghe et al [21] analyzed the evolution of the infection using parasitological methods in 29 naturally infected rodents. The mammals belonged to four species: *Sigmodon hispidus* (2), *Oryzomys melanotis* (12), *Ototylomys phyllotis* (9) and *Peromyscus yucatanicus* (6). In a second study [22], infection by *Leishmania mexicana* was detected in eight mammal species using two methods – in culture and PCR. The *Leishmania* parasite was confirmed by both methods in six species: *O. phyllotis*, *Heteromys gaumeri*, *O. melanotis*, *P. yucatanicus*, *S. hispidus*, and *Heteromys desmarestianus*. In the other two species it was confirmed using only via one of the methods: in culture for *Marmosa mexicana* and by PCR for *Reithrodontomys gracilis*.

As collection data is fundamentally tied to a taxonomic classification, it is natural to describe the biota in terms of taxa and consider the spatio-temporal distribution of a species for example. For a data set that covers a spatial area A and time interval T one may divide the space and interval into spatio-temporal cells, (x_α_, t_β_) which form a mesh that partitions both the geographic region and time interval. The labels x_α_ and t_β_ simply indicate the particular spatio-temporal cell we are considering. A point collection associated with this cell is such that it corresponds to a latitude and longitude within the spatial cell x_α_ and to a collection date in the temporal cell t_β_. We can consider the distribution of the set of species, B(x_α_, t_β_) = (B_1_(x_α_, t_β_), … B_NB_(x_α_, t_β_)), where B_i_(x_α_, t_β_) is a measure of the distribution of the ith taxon in a spatial cell x_α_, in the time interval t_β_. A natural realization of B_i_ (x_α_, t_β_) would be the abundance of the taxon i in the spatial cell x_α_, in the time interval t_β_ as measured by its frequency or relative frequency. A less discriminating realization for B_i_ (x_α_, t_β_) would be a function that indicates only presence or presence/absence in the geographic region x_α_ in the time interval t_β_. As B_i_ (x_α_, t_β_) is a stochastic variable, the distribution of any taxon B_i_(x_α_, t_β_) is described by a probability distribution, P(B_i_(x_α_, t_β_)), whose evolution, in principle, depends on both biotic factors, B_j_(x_ρ_, t_σ_), associated with other species, and abiotic factors, A(x_ρ_, t_σ_) = (A_1_(x_ρ_, t_σ_), …, A_NA_(x_ρ_, t_σ_))_,_ such as temperature, precipitation etc., where we consider cells x_ρ_, t_σ_ that may be different to a given cell x_α_, t_β_ to indicate that, in principle at least, there may be statistical associations between a given spatio-temporal cell and others. The full ecological niche at x_α_ and t_β_ can be described by a vector **I**(x_α_, t_β_) = (A_1_ (x_α_, t_β_),…, A_NA_ (x_α_, t_β_); B_1_(x_α_, t_β_),…, B_NB_ (x_α_, t_β_)).

A full model would consist of determining P (B_i_(x_α_, t_β_)) = F (**I** (x_ρ_, t_σ_)), relating the distribution of a subset of biota at one place and time to all biotic and abiotic factors considered at different places and times. Of course, there are no underlying fundamental principles on which to build the function F. We therefore adopt a non-parametric “data mining” approach, modeling the distribution directly using available data, rather than constructing an a priori parametric model. An advantage of this approach is that the observed distribution is a direct result of the past and present interactions of all relevant causative factors - climactic, phylogenetic, co-evolutionary, ecological etc. Nothing is omitted. However, an observation of P (B_i_(x_α_, t_β_)) in itself does not provide a predictive model. To create such a model we consider the problem as a classification task, relating a class, such as the class of cells with presence of a given species, to a feature vector **I** using the conditional probabilities P (B_i_| **I**). Converting the problem to one of classification is very natural from the point of view of presence or presence/absence. In the case of abundance a coarse graining of the abundance data in a given spatial-temporal cell is required. This can be achieved in many ways, depending on how many classes are posited and the criterion by which a given abundance fits in a given category. For example, one might classify abundance into three categories – Low, Normal and High – where Low is any abundance at least one standard deviation below the average and High is any abundance at least one standard deviation above the average. One can then naturally consider the conditional probability that a High abundance of species B_i_ is found given a High abundance of species B_j_. Of course, in order to do this, one requires abundance data in the first place. As this is less common than presence or presence/absence data, and simply not available in the context of emerging diseases such as Leishmaniasis, we will here focus on the latter. For the same reason, in the following, we will also restrict attention to the spatial dependence of the distributions and ignore the temporal aspect, as the data simply is not capable of reliably describing temporal changes.

The class, we will take to be a taxon distribution, B_i_, while the feature vector set is taken to be a subset of niche variables 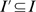. In this case, **I** ^′^, represents a niche profile with both biotic and abiotic components which constitute the biotic and abiotic profiles of the niche. For a given taxon, 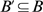, and niche variables, 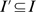, our chief object of study is the probability P(B_i_ | **I**'^′^) = N_BiAND I_′/N_I_′, where N_BiAND I_′ is the number of spatial cells where there is a co-occurrence of the taxon B_i_ and the niche variables **I** ^′^, and N_I_′ is the number of cells where the niche variables take their stated values. The niche profile **I** ^′^(x_α_) associated with a spatial cell x_α_ then determines the probability of the distribution variable, B_i_(x_α_), in that cell, and one now has a predictive model. Note that, although we concentrate on biotic variables in the present paper, in the current approach, all niche variables can be treated on a democratic footing. The problem of calculating P (B_i_ | **I′**) directly is that both N_Bi AND I_′ and N_I_′ are likely to be zero when the number of taxa or niche variables considered simultaneously is large, as there will tend to be no co-occurrences of so many variables. This can be ameliorated by considering a reduced number of both class and feature variables. For instance, P (B_i_ | **I**_k_) is determined by the number of co-occurrences of the taxon B_i_ and the niche variable **I**_k_ and, in principle, allows us to find the most important statistical associations between the niche variables and the taxa distributions. However, P(B_i_ | **I**_k_) being a probability does not account for sample size. For example, if P(B_i_ | **I**_k_) = 1 this may be as a result of there being a coincidence of B_i_ and **I**_k_ in one spatial cell or 1,000. Obviously, the latter is more statistically significant. To remedy this we consider the following test statistic
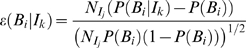
(1)which measures the statistical dependence of B_i_ on **I**_k_ relative to the null hypothesis that the distribution of B_i_ is independent of **I**_k_ and randomly distributed over the grid, i.e., 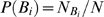, where 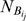 is the number of grid cells with point collections of species B_i_ and N is the total number of cells in the grid. The sampling distribution of the null hypothesis is a binomial distribution where, in this case, every cell is given a probability P(B_i_) of having a point collection of B_i_. The numerator of equation (1) then, is the difference between the actual number of co-occurrences of B_i_ and I_k_ relative to the expected number if the distribution of point collections were obtained from a binomial with sampling probability P(B_i_). As we are talking about a stochastic sampling the numerator must be measured in appropriate “units”. As the underlying null hypothesis is that of a binomial distribution, it is natural to measure the numerator in standard deviations of this distribution and that forms the denominator of equation (1). In general, the null hypothesis will always be associated with a binomial distribution as in each cell we are carrying out a Bernoulli trial (“coin flip”). However, the sampling probability can certainly change. For instance, one could take as null hypothesis a binomial distribution with sampling probability P(B_i_|M = 1) = N_Bj_/N_M = 1_, where M here is a binary variable associated with the fact that a niche-variable model, such as GARP or MaxEnt, says whether the species B_i_ is present or absent. N_M_ is then the number of cells where the niche model says there is presence. Taking P(B_i_|B_k_,M) relative to the null hypothesis P(B_i_|M) tells us how the presence of species B_j_ is associated with the presence of B_k_ in the context of cells where a niche model has indicated the presence/absence of B_k_. In other words, how B_k_ affects the distribution of B_i_ in those places where the niche model says B_k_ is present/absent.

The quantitative values of ε(B_i_ |B_k_) can be interpreted in the standard sense of hypothesis testing by considering the associated p-value as the probability that |ε(B_i_ |B_k_)| is at least as large as the observed one and then comparing this p-value with a required significance level. In the case where 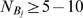 then a normal approximation for the binomial distribution should be a decent approximation and in this case ε(B_i_ |B_k_) = 2 would represent the standard 95% confidence interval. In the case where a normal approximation is not accurate then other approximations to the cumulative probability distribution of the binomial must be used.

In the case where **I**_k_ = B_k_, another taxon, then P(B_i_ |B_k_) and ε(B_i_ |B_k_) are measures of the statistical association between the two taxa, ε(B_i_ |B_k_) having the added advantage of having built into it the degree of statistical confidence that one may have about the association. Note that such a statistical association does not necessarily prove that there is a direct “causal” interaction between the two taxa. Rather, it allows for a statistical inference that may be validated subsequently.

From either P(B_i_ |B_k_) or ε(B_i_ |B_k_), an inferential interaction network between taxa can be constructed where the nodes are the taxa and the links represent the degree of statistical dependence of one on the other. The links must represent the degree of interaction as otherwise one has a uniform fully connected network. This can be done, for instance, by only showing the principle interactions above a certain threshold of ε or P, or by having the link width or size depend on their values. Note that such an interaction network, being based on point collection data, is inferential with respect to real biotic interactions between the taxa. This is distinct to other networks where network links are determined observationally. P (B_i_ |B_k_) and ε(B_i_ |B_k_) are measures of pair-wise dependencies between taxa. They can be generalized to take into account higher order interactions. For instance, ε(B_i_ |B_k_ B_m_) measures the statistical interaction between the joint presence of taxa B_m_ and B_k_ and that of taxon B_i_.

Probabilities P (B_i_ |**I** ^′^), where **I** ^′^ is of high dimension, can be constructed using different classification models, such as neural networks, discriminant analysis etc. A particularly transparent, simple and effective approximation is the Naive Bayes approximation [26] with
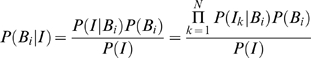
where, in the first equality, Bayes rule has been used, and in the second it has been assumed that the niche variables I_k_ are independent. The product here is over the N niche variables under consideration as conditioning factors for B_i_. In the case of the relationship between *Lutzomyia* and mammals, N represents the number of mammal species. A score function that can be used as a proxy for P (B_i_ |**I** ^′^) is
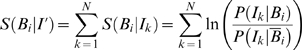
where 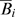 is the complement of the set B_i_. For example, if B_i_ is the set of cells with presence of taxon B_i_ then 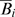 represents the set of cells without presence. S(B_i_ | **I** ^′^) is a measure of the probability to find the distribution variable B_i_ when the niche profile is **I** ^′^. It can be applied to a spatial cell x_α_ by determining the niche profile of the cell, **I** ^′^(x_α_). As an example, for two biotic niche variables, B_2_ and B_3_, that take values 1 (corresponding to the fact that there is a point collection associated with that cell) and 0 (there is no point collection associated with the cell), the four possible biotic niche profiles of any cell are (B_2_, B_3_) = (0,0); (0,1), (1,0) and (1,1). The score contributions of each biotic variable are S(B_i_|B_2_) and S(B_i_|B_3_), calculated using the above formula. Hence, S(B_i_ | **I** ^′^) = S(B_i_ | B_2_, B_3_) = S(B_i_|B_2_)+S(B_i_|B_3_). Thus, for any given spatial cell x_α_ one can assign a niche profile, i.e. values of B_2_ and B_3_, from whence it is possible to assign a corresponding score. If there is no statistical association between B_i_ and B_2_ or B_3_ then the corresponding score contributions are zero. An overall zero score then signifies that the probability to find B_i_ js the same as would be found if B_i_ were distributed randomly. If the score is positive then there is a higher than random probability to find B_i_ present and on the contrary if the score is negative.

The geographical region of interest for the data of the present study is Mexico. Within this specified region there is an important question of how to choose an appropriate mesh size. The right degree of coarse graining is essentially governed by the size of the data set available relative to the data necessary to construct a given probability function. For instance, to calculate P(B_i_, B_k_), where B_i_ represents presence of species i in grid cell x_α_ : If the mesh size is too small then the probability of a co-occurrence of species i and k is very small. On the other hand, if the mesh size is too big then, as well as a lack of statistical significance, discrimination will also be lost. A reasonable estimate of the appropriate cell size can be determined by assuming that the N collections are distributed randomly in an area A. An appropriate cell size is then A^1/2^/N, which corresponds to having, on average, one collection per cell. Given that we are emphasizing here pairwise associations between species, the appropriate value of N is the average number of collections for any species. A more sophisticated methodology is to consider the number of co-occurrences as a function of cell size and look for the maximum of this function. This can be done for a particular pair of species, or one may consider an average over different pairs. For our study we used 3,337 square cells of linear size 25 km which corresponds to an average number of point collections of about 20.

Checks were made with other cell sizes of 5 km, 10 km, 50 km and 100 km to assure the robustness of our conclusions. In Table 2, for the ranked list of potential reservoirs we see how the average position in the ranked list changes as a function of cell size. This shows that the relative ranking is quite insensitive to the cell size, as the z-scores of the average rank of six of the known reservoirs relative to the expected average rank if the distribution were random are highly statistically significant. In other words, the predictions as to which species are most likely to be reservoirs are robust to large changes in the cell size. In general, the absolute values of epsilon will change as a function of cell size, principally due to the effect of reducing the number of co-occurrences as one passes to large cell sizes or to very small cell sizes. However, relative values of epsilon will remain quite stable.

**Table 2:** Relative rank by score of known reservoirs for *Leishmania* in Mexico as a function of grid size.

Species	5 km	10 km	25 km	50 km	100 km
Didelphis marsupialis	52	31	40	17	22
Heteromys gaumeri	1	13	6	47	38
Sigmodon hispidus	17	19	27	50	90
Ototylomys phyllotis	2	5	22	60	40
Oryzomys melanotis	90	54	88	72	51
Peromyscus yucatanicus	3	10	28	84	62
Average Rank	27.50	22.00	35.17	55.00	50.50
z-score	−12.54	−25.93	−15.48	−16.69	−16.91
